# Frontiers in Biophotonics

**DOI:** 10.1117/1.JBO.31.11.113501

**Published:** 2026-11-12

**Authors:** Katarzyna Komolibus, Gavrielle R. Untracht, Stefan Andersson-Engels, Peter E. Andersen

**Affiliations:** a Tyndall National Institute, Biophotonics@Tyndall, Cork, Ireland; b Technical University of Denmark, Department of Health Technology, Kongens Lyngby, Denmark; c University College Cork, School of Physics, Cork, Ireland

## Abstract

Biophotonics is poised to play a central role in the transition toward personalized, data-driven healthcare by enabling increasingly sensitive, quantitative, and minimally invasive measurements of biological systems. This editorial examines interconnected frontiers shaping the field, and highlights how advances across these areas can collectively accelerate clinical translation. Its perspective emphasizes education, interdisciplinary collaboration, and the development of future researchers as the foundation for converting technological progress into meaningful healthcare impact.

## 1 Introduction

The healthcare landscape is rapidly evolving towards real-time, minimally invasive diagnostics, while the era of one-size-fits-all treatment is gradually becoming a thing of the past. As biotechnology and pharmacology continue to expand the range of available diagnostics and interventions, the challenge of reliably determining which treatment will benefit a given patient is becoming even more prominent. Advances in genomic sequencing, molecular profiling, and artificial intelligence (AI) offer the potential to tailor treatment to the characteristics of each individual and their disease rather than to a broad diagnostic category.^1^ The appeal is obvious: patients stand to gain more targeted treatment with fewer side effects, while health systems could make more efficient use of resources through earlier and more precise interventions.

Biophotonics has a key role to play in this transition. Optical technologies have transformed the way we detect, monitor, and treat diseases.^2^ They can probe tissue composition, structure, and function non-destructively and, in many cases, without exogenous labels. This sensitivity to biochemical, functional, and morphological contrast, across scales ranging from subcellular organelles to whole organs, is difficult to obtain by any other modality. As light-based technologies mature from laboratory demonstrations towards clinical tools, they offer exactly the type of quantitative, information-rich data that personalized medicine increasingly requires.^3^

Realizing this promise will depend on progress across several frontiers in biophotonics simultaneously. In this editorial, we consider four interconnected frontiers of biophotonics: performance, access, reach, and intelligence (Fig. 1). “Performance” concerns measurable capabilities such as sensitivity, resolution, imaging depth, acquisition speed, and molecular specificity. “Access” describes affordability, usability and deployment across clinical and resource-limited settings. “Reach” refers to the tissue sites and biological processes that optical systems can interrogate. “Intelligence” concerns the extraction of clinically relevant information from complex measurements through computational analysis and AI, together with the standardization required to make data trustworthy and comparable. These categories are complementary and overlapping, but together they provide a framework for considering both near-term and longer-term opportunities in biophotonics.

**Fig. 1:**
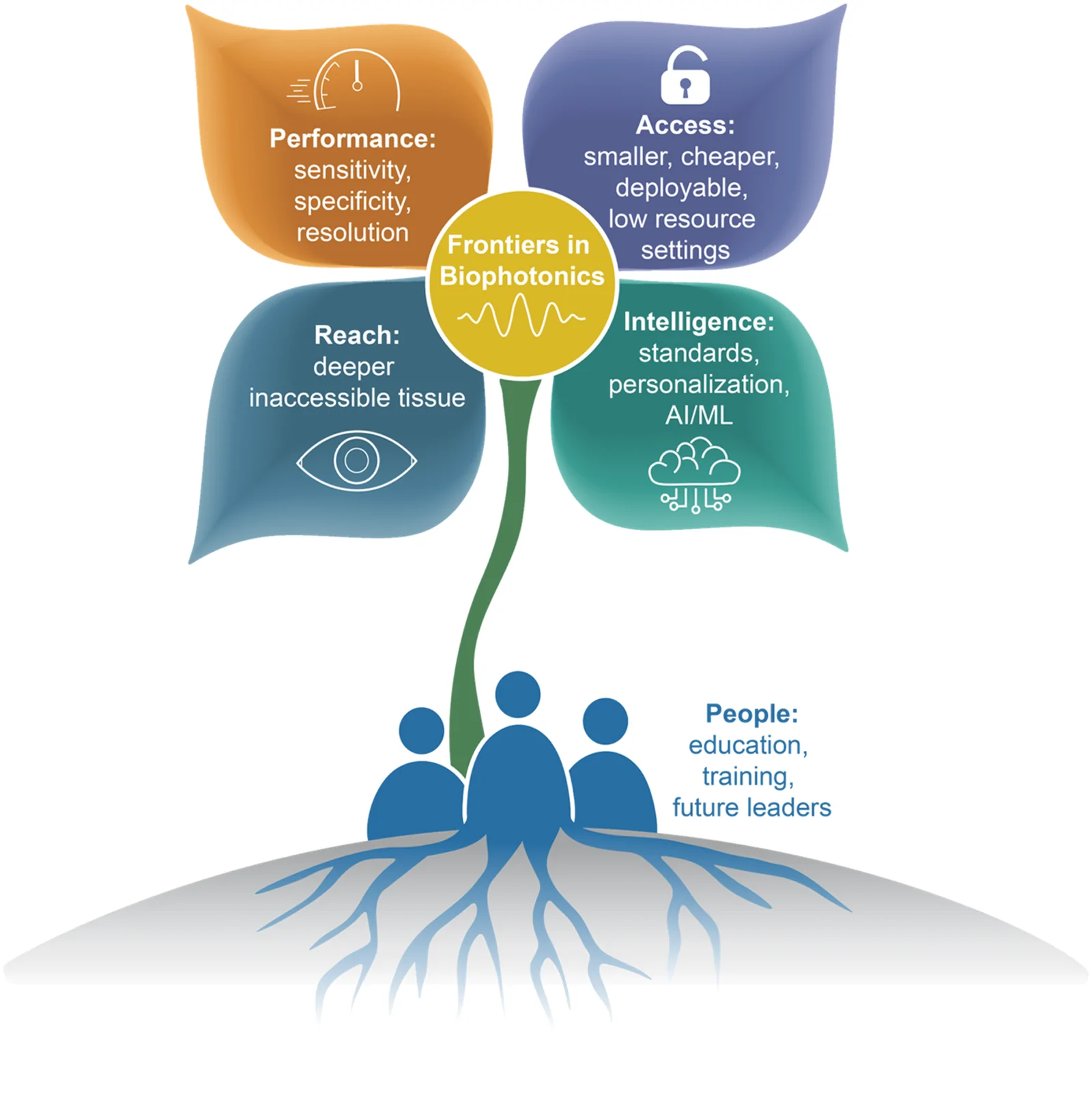
Four interconnected frontiers in biophotonics—performance, access, reach and intelligence—resting on a foundation of people.

Advancing these interconnected frontiers depends not only on technological innovation, but also on the people who develop, evaluate, and apply these technologies. Training and education of the next generation are therefore the foundations for all four frontiers rather than separate dimensions. This is one reason why initiatives like the SPIE Biophotonics Summer School^4^ are important and increasingly relevant. For more than 20 years, this summer school has provided a platform for education and community building, connecting young researchers with their peers and key leaders in the field. Through training the next generation of researchers, we can build a strong and collaborative community that will advance the field and positively impact healthcare. This JBO special section marks the 12th edition of the summer school.

## 2 Special Collection

The contributions collected in this special section demonstrate the progress already being made across these interconnected frontiers. Advances in single-photon detection^5^ and in spatio-temporal optical coherence imaging^6^ extend performance and reach; point-of-care biophotonics for low-resource settings^7^ boost access; solid-state fluorescence-lifetime standards^8^ address the standardization on which trustworthy, AI-ready data depend; and experiential education^9^ sustains the people who underpin developments across all four frontiers.

Building on these contributions, the subsequent sections of this editorial explore selected developments and emerging opportunities across the four frontiers. The examples that follow are chosen to be representative rather than providing a comprehensive overview of biophotonics. They deliberately span different stages of maturity, from technologies already established in biomedical research or selected clinical settings to emerging concepts that remain under development.

## 3 Performance

Historically, advances in biophotonics have been closely linked to developments in enabling technologies, particularly light sources and detector systems. A classic example is multiphoton fluorescence microscopy. Although multiphoton excitation was first demonstrated in 1961,^10^ its application to biological microscopy only became practical once laser technology had matured sufficiently to provide the high peak powers and appropriate wavelengths required for biological imaging.^11^ This interplay between technological innovation and scientific discovery continues to drive the field: advances in the underlying photonic technologies enable new measurement capabilities, which in turn allow previously inaccessible biological and clinical questions to be addressed.

Detector and source technologies sit at the performance frontier, driving the pursuit of greater sensitivity, resolution, imaging depth, acquisition speed, and molecular specificity. Detector systems are becoming progressively faster and more sensitive, while simultaneously moving from specialized laboratory components towards increasingly practical imaging technologies. Single-photon avalanche diode (SPAD) arrays, for example, combine single-photon sensitivity with precise temporal information and are enabling new approaches to time-resolved sensing/imaging, light-sheet fluorescence lifetime imaging, and super-resolution microscopy.^12^ At the limits of photon detection, superconducting nanowire single-photon detectors (SNSPDs) offer exceptionally low noise and picosecond-scale temporal resolution. Such capabilities are particularly promising for applications in which photon budgets are intrinsically limited, including deep-tissue fluorescence imaging and emerging quantum-enhanced approaches as discussed within this special section by Hughes et al.^5^ (featured on the issue cover). Collectively, these developments extend the range of signals that can be detected and the biological dynamics that can be observed.

A parallel evolution is taking place in light-source technology. Sources operating in the short-wave infrared (SWIR) or NIR-II region are of particular interest because tissue scattering generally decreases with increasing wavelength, enabling improved spatial resolution and image contrast at depth when appropriate spectral windows with sufficiently low tissue absorption are selected.^13^^,^^14^ Such sources are already being explored in modalities including optical coherence tomography and photoacoustic imaging.^15^ In parallel, efficient ultrafast sources in the 1300 to 1700 nm range are driving advances in nonlinear microscopy, including three-photon fluorescence microscopy.^16^^,^^17^ Looking further ahead, quantum-enabled sources generating non-classical states of light, including squeezed light and entangled photon pairs, offer routes towards enhanced measurement sensitivity at a given optical exposure, which is particularly attractive for biological imaging where photodamage can fundamentally limit measurement performance.^18^^,^^19^ Thus, progress at the level of individual photonic components continues to push fundamental limits in sensitivity, resolution, imaging depth, and information content.

## 4 Access

Progress towards clinical translation, however, is not defined solely by improvements in the performance of laboratory instruments. An equally important frontier is access: the transition from complex, laboratory-based instrumentation towards compact, robust, and increasingly affordable systems. Biophotonics technologies typically emerge from sophisticated optical arrangements requiring substantial space, controlled environments, and expert operators. Such systems remain indispensable for developing new concepts and understanding fundamental light–tissue interactions, but their complexity can become a major barrier to clinical translation. Advances in photonic and semiconductor integration, together with progress in micro-optics, fiber optics, compact sources and detectors, and embedded electronics, are enabling increasingly miniaturized, robust, and integrated biophotonic systems.^20^ Functions that previously required an optical table can increasingly be incorporated into handheld, endoscopic, wearable, or even chip-scale devices. Designing systems around robustness, affordability, and ease of use can move advanced optical sensing and imaging from specialized laboratories towards outpatient clinics, primary care, the bedside, and ultimately everyday environments.

Miniaturization and integration have another important consequence: they change how often and in what settings measurements can be performed. Wearable technologies are particularly interesting in this respect because they shift biophotonics from isolated measurements towards longitudinal or continuous observations. Rather than providing a single snapshot of physiology or disease, such devices could reveal trajectories of change and responses to interventions over time, generating new forms of data whose interpretation becomes part of the intelligence frontier discussed below.

Affordability and simplicity are equally important for extending access geographically and societally.^18^ Compact optical systems, particularly when combined with smartphones, low-cost semiconductor components, and automated data analysis, may provide sophisticated diagnostic capabilities in resource-constrained environments where conventional laboratory or imaging infrastructure is unavailable.^21^ This aspect of the access frontier is examined within this special section by Mthunzi-Kufa et al. from the South African perspective.^7^ Importantly, this should not be regarded simply as producing simplified versions of technologies originally developed for high-resource healthcare systems. Designing from the outset for affordability, robustness, ease of use, and minimal supporting infrastructure can itself stimulate innovation, with benefits that extend across healthcare settings.

## 5 Reach

Emerging capabilities in miniaturization also shift where optical measurements can be made, expanding the reach frontier. Much of biomedical optics has inevitably been shaped by the anatomical locations accessible with existing instrumentation. Miniaturized cameras, fiber-optic probes, chip-scale sensors, and integrated photonic devices can increasingly be incorporated into endoscopes, catheters, needles, and other minimally invasive instruments.^20^^,^^22^ This creates opportunities to obtain structural, functional, and molecular information directly from deep or confined tissue sites that have previously been difficult or impossible to interrogate optically.

Beyond anatomical accessibility, reach also concerns the range of biological processes that optical technologies can interrogate. Advances in sensitivity, spectral coverage, temporal resolution, and molecular specificity can make previously inaccessible biochemical, metabolic, and dynamic processes observable. In addition to hardware improvements, new measurement concepts and methodologies can translate progress in enabling technologies into new imaging capabilities. Spatio-temporal optical coherence imaging (STOC), reviewed within this special section by Wojtkowski,^6^ illustrates how advances in imaging performance can extend biological reach. By improving imaging fidelity in scattering media, STOC enables high-resolution and high-contrast visualisation of structures that would otherwise be difficult to investigate *in vivo*. This example demonstrates how novel technologies can open new avenues for biological investigation, while emerging applications demand further technological innovation. Together, they provide opportunities for earlier, less invasive diagnostics and for observing biological processes that were previously not accessible *in vivo*, ultimately expanding the biological and clinical questions that can be addressed.

The distinction between anatomical and biological reach also highlights the interaction between reach and access frontiers. A miniaturized optical system may simultaneously increase reach by allowing measurements within a previously inaccessible organ and increase access by reducing the size, complexity, or infrastructure required for its deployment. The four frontiers should therefore not be viewed as independent technological pathways, but as interconnected dimensions along which individual innovations can drive progress simultaneously.

## 6 Intelligence

The fourth frontier concerns not only how measurements are acquired, but how they can be converted into clinically meaningful information. The rapid maturation of AI offers more powerful ways to extract clinically relevant information and predictions from complex, high-dimensional measurements. Yet the performance and trustworthiness of machine-learning models depend fundamentally on the quality of the data on which they are trained.^23^ Larger multicenter databases will be required for clinical translation, but scale alone is not sufficient. Robust and generalizable models also depend on measurements acquired across different instruments, sites, and patient cohorts, to be mutually comparable. AI alone cannot compensate for inconsistent and insufficiently reproducible measurements. Standardization is therefore an important prerequisite for robust and generalizable insights from biophotonic data.

The tools needed to address this challenge have advanced considerably. Tissue-mimicking phantoms have become increasingly common.^8^^,^^24^ The solid-state fluorescence lifetime phantoms reported within this special section by Angelone et al.^8^ highlight how such tools can support reproducible and comparable measurements. Furthermore, individual subfields are beginning to converge on measurement protocols and standards, with recent consensus guidelines for label-free optical metabolic imaging providing one example.^25^ Nevertheless, such efforts remain fragmented and largely modality-specific, and broader standardization of the field will be necessary as we move towards multicenter datasets and AI-assisted decision processes.

The convergence of miniaturized and wearable sensors, longitudinal measurements and AI creates additional opportunities for generating insights into physiology and disease over time and providing datasets fundamentally different from conventional single-time-point observations. Looking further ahead, such measurements may also contribute to the development of patient-specific digital twins, in which repeated optical measurements inform and update computational representations of tissue physiology, disease state, or treatment response.^26^ Biophotonics could thus provide an important bridge between the physical patient and an evolving digital representation, allowing computational models not only to describe the current state of tissue but ultimately to predict its future behaviour or response to intervention. In this context, the combination of quantitative optical measurements, longitudinal monitoring, and computational modelling may contribute to the broader transition from reactive healthcare towards increasingly preventive, predictive, and personalized medicine.

## 7 From Technological Frontiers to Clinical Impact

The four frontiers provide a framework for understanding where biophotonics is advancing; their convergence determines how this progress may translate into clinical and societal impact. Greater sensitivity, resolution, imaging depth, and molecular specificity remain fundamental goals, but they must be accompanied by broader access to deployable technologies, greater anatomical and biological reach, and trustworthy interpretation of resulting data. Importantly, advances along one frontier can accelerate developments along another. Figure 2 highlights how these interconnected frontiers may collectively contribute to healthcare impact. Rather than representing a second conceptual framework, it illustrates three complementary translational trajectories towards personalized, data-driven care: from laboratory instrumentation towards deployable devices; from specialized healthcare environments towards measurements increasingly closer to the patient; and from raw optical measurements towards standardized data, computational interpretation, and clinical decision support. These trajectories are not independent of the four frontiers in Fig. 1 but instead illustrate how their interplay can facilitate the transition from technological capability towards clinical utility.

**Fig. 2:**
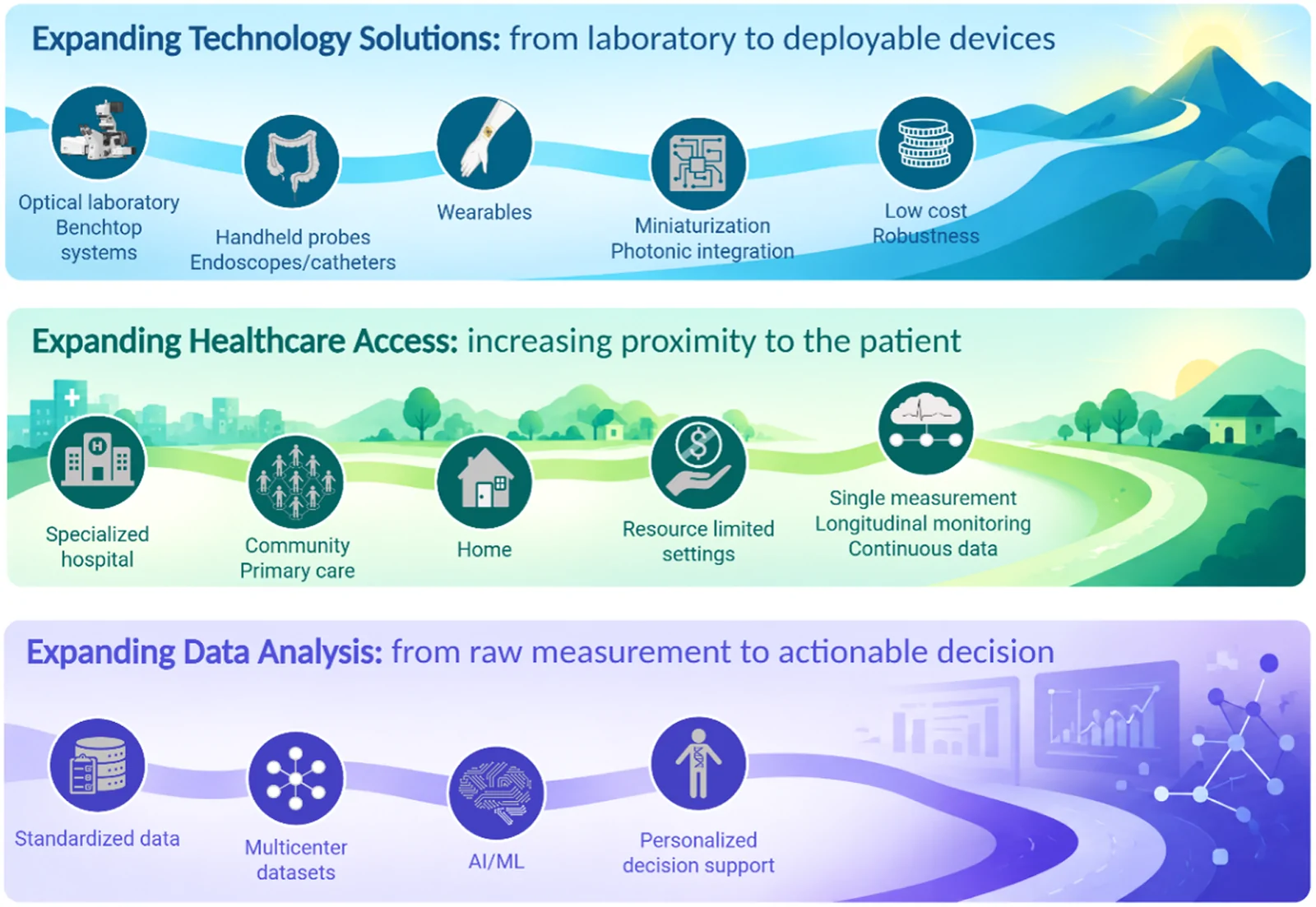
Translational roadmap in biophotonics: advances across the four frontiers shown in Fig. 1 converge along three broad pathways towards personalized, data-driven care. Created in BioRender.^27^

The future impact of biophotonics will therefore depend not on progress along any one frontier in isolation, but on their convergence: improved performance can extend biological reach, technological innovation can broaden access, and increasingly rich measurements must be transformed through standardization and intelligence into information that can guide clinical decisions.

## 8 People—the Foundation of the Four Frontiers

Technological advances alone, however, will not determine how far these frontiers can be pushed. Biophotonics is inherently interdisciplinary, drawing on optics and photonics, engineering, chemistry, biology, medicine, data science, and increasingly AI. Progress therefore depends on researchers who are not only highly trained within their own disciplines, but who can also communicate and work effectively across disciplinary boundaries.

Education in biophotonics has an important role in developing this breadth. Students need a strong grounding in the underlying principles of light–tissue interaction and optical instrumentation, while also learning to understand biological questions, clinical needs, and the practical constraints that determine whether a promising technology can ultimately be translated. The role of experiential education and seasonal schools in building this breadth is discussed within this special section by Mthunzi-Kufa et al.^9^ Initiatives such as the SPIE Biophotonics Summer School^4^ can bring together education, mentorship, interdisciplinary exchange, and collaboration, while complementary programs across institutions and regions are needed to broaden participation and prepare researchers to connect physical principles, biological questions, engineering constraints, and clinical needs.

Equally important is the development of the future scientific leaders of the field. Training environments that bring together students, early-career researchers, established scientists, clinicians, and industry can provide something that conventional disciplinary education alone cannot: exposure to different ways of framing problems and opportunities to build networks across institutional and national boundaries. Open access to educational material authored by leaders in the scientific field for educating next-generation scholars is imperative and requires visionary organizations to curate and provide such material.^28^ As the technological frontiers of biophotonics continue to expand, cultivating researchers capable of connecting these frontiers and translating advances between physics, chemistry, engineering, biology, and medicine will be essential for converting technological possibility into meaningful scientific and clinical impact.
